# GBP2 as a potential prognostic predictor with immune-related characteristics in glioma

**DOI:** 10.3389/fgene.2022.956632

**Published:** 2022-09-16

**Authors:** Ren Li, Yuan-Yuan Wang, Shu-Le Wang, Xue-Peng Li, Yang Chen, Zi-Ao Li, Jian-Hang He, Zi-Han Zhou, Jia-Yu Li, Xiao-Long Guo, Xiao-Gang Wang, Yong-Qiang Wu, Ye-Qing Ren, Wen-Ju Zhang, Xiao-Man Wang, Geng Guo

**Affiliations:** ^1^ Department of Neurosurgery, The First Hospital of Shanxi Medical University, Taiyuan, Shanxi, China; ^2^ Department of Radiology, the First Affiliated Hospital, Zhejiang University School of Medicine, Hangzhou, Zhejiang, China; ^3^ Department of Vascular Surgery, The Second Hospital of Shanxi Medical University, Taiyuan, Shanxi, China; ^4^ Department of Biochemistry and Molecular Biology, Institute of Basic Medical Sciences, Chinese Academy of Medical Sciences and Peking Union Medical College, Beijing, China

**Keywords:** gbp2, glioma, prognosis, immune infiltration, non-coding RNA

## Abstract

Guanylate binding protein 2 (GBP2) is a member of the guanine binding protein family, and its relationship with prognostic outcomes and tumor immune microenvironments in glioma remains elusive. We found GBP2 were increased in glioma tissues at both mRNA and protein levels. Kaplan-Meier curves revealed that high GBP2 expression was linked with worse survival of glioma patients, and multivariate Cox regression analysis indicated that high GBP2 expression was an independent prognostic factor for glioma. Combined analysis in immune database revealed that the expression of GBP2 was significantly related to the level of immune infiltration and immunomodulators. Single-cell analysis illustrated the high expression of GBP2 in malignant glioma cells showed the high antigen presentation capability, which were confirmed by real-time polymerase chain reaction (qRT-PCR) data. Additionally, the hsa-mir-26b-5p and hsa-mir-335-5p were predicted as GBP2 regulators and were validated in U87 and U251 cells. Our results first decipher immune-related characteristics and noncoding regulators of GBP2 in glioma, which may provide insights into associated immunotherapies and prognostic predictor.

## Introduction

Gliomas are common primary intracranial tumors from the neuroepithelium, accounting for 81% of central nervous system (CNS) malignancies. Gliomas usually originate from glial cells or precursor cells and gradually develop into astrocytoma, oligodendroglioma, ependymoma, or oligoastrocytoma (Zhang et al., 2012; Ostrom et al., 2018). At present, the World Health Organization (WHO) divides gliomas into four grades. Grades 1 and 2 gliomas represent low grade with the median survival time of 11.6 years, and grades 3 and 4 gliomas are high grade (Ohgaki and Kleihues, 2005; Louis et al., 2016) with an overall survival (OS) time of approximately 15 months to 3 years (Bleeker et al., 2012).

At present, the main strategy for glioma treatment is surgical resection, especially maximal surgical resection (Hervey-Jumper and Berger, 2016; Patil et al., 2019). Surgical treatment of low-grade gliomas has the potential to lead to disease recovery, while high-grade gliomas are incurable (de Leeuw and Vogelbaum, 2019). For high-grade gliomas, temozolomide is usually used for chemotherapy. The use of temozolomide in animal models improves survival and reduces tumor volume. Although temozolomide can help glioma patients to prolong survival to some extent, it is prone to develop resistance (Hirst et al., 2013; Weil et al., 2017; Karachi et al., 2018). The Electric field therapy can be used for gliomas that are difficult to operate or recur early, while the cost of treatment is expensive (Mun et al., 2018). Gliomas can currently be treated with surgery, radiotherapy, and chemotherapy, but the combinational therapy do not completely remove all tumor cells, which lead to high recurrence rate and the low overall survival rate (Weil et al., 2017; Xu et al., 2020). Recently, treatment of glioma by immune targets has emerged as a potential new therapeutic strategy. Combined blockade of IL-12 and CTLA-4 resulted in a dramatic decrease in FoxP3+ T reg cells and an increase in effector T (T eff) cells to achieve clearance of glioblastoma (Vom Berg et al., 2013). PD-1/PD-L1 checkpoint blockade also has potential for the treatment of glioblastoma (Wang et al., 2019). Thus, investigating the immune-related mechanisms would facilitate the development of immunotherapy in glioma treatment.

Guanylate binding protein 2 (GBP2) is a member of the guanine binding protein (GBP) family that can bind to guanine nucleotides (GMP, GDP, and GTP) (Tretina et al., 2019). GBP2 has been reported to increase glioblastoma invasiveness through the Stat3/fibronectin pathway (Yu et al., 2020). GBP2 has also been implicated in the prognosis of pancreatic cancer, cutaneous melanoma, and is involved in cutaneous melanoma progression through the Wnt/beta-catenin pathway (Ji et al., 2021; Liu et al., 2021). Our previously study showed that GBP2 could regulate the cell growth and migration of glioma cells (Ren et al., 2022). However, there are fewer studies on the immune properties of glioma related to GBP2. In this study, we examined the clinical relevance of GBP2 to glioma and explored the immune properties of GBP2 using single cell, immune infiltration and immunomodulatory analyses, and finally analyzed potential regulators of GBP2. We therefore investigated the effect of GBP2 on glioma prognosis and explored the immune-related mechanisms, hoping to deepen the understanding of potential immunotherapeutic target in glioma.

## Materials and methods

### Data collection

RNA-seq data for glioma were obtained from the Cancer Genome Atlas (TCGA) (https://portal.gdc.cancer.gov/). TCGA Pan-Cancer and GTEx normal tissues TPM data were obtained from UCSC Xena (https://xenabrowser.net/datapages/) (Vivian et al., 2017). RNA-seq data were processed through TOIL. All samples were normalized and filtered using the statistical programming language R and the R package “limma.” The result was visualized using the R package “ggplot2.”

### Clinical relevance analysis of guanylate binding protein 2

Glioma patients were divided into two groups according to the median GBP2 high and low expression and analyzed with R software. The association between clinical variables and GBP2 expression was analyzed with R software (Ceccarelli et al., 2016). Wilcoxon rank sum test was used to compare two groups, and Kruskal-Wallis test was used to compare multiple groups. To determine the predictive power of GBP2 expression in clinical variables, we further plotted receiver operating characteristic curve (ROC) with the package “pROC” (21). We used logistics regression analysis to speculate the correlation between clinical variables and GBP2 (22).

### GEPIA: Gene Expression Profiling and Interactive Analyses and UALCAN database analysis

The GEPIA database (http://gepia.cancer-pku.cn/index.html) (Tang et al., 2017) was used to further analyze the differences in mRNA expression levels of GBP2 between glioma and normal tissues. |Log_2_FC| Cutoff was 1 and *p*-value Cutoff was 0.01. The UALCAN database (http://ualcan.path.uab.edu/index.html) is a website to analysis gene expression data, which from TCGA. (Chandrashekar et al., 2017) We analyzed GBP2 protein expression level in pan-cancer with it. Statistical significance: ns, *p* ≥ 0.05; *p < 0.05; **p < 0.01; ***p < 0.001.

### The human protein atlas database analysis (immunohistochemistry)

The Human Protein Atlas (HPA) database (https://www.proteinatlas.org/) is a website that provides the distribution of proteins in human tissues and cells. The website uses immunohistochemical techniques to detect the expression of various proteins in normal and tumor tissues (Sjostedt et al., 2020). The results are expressed by immunohistochemical staining maps and read and indexed by professionals. We utilized The Human Protein Atlas database for GBP2 protein expression differences between glioma tissue and normal brain tissue.

### Prognostic analysis of guanylate binding protein 2

Kaplan-Meier (KM) curve and univariate/multivariate Cox regression were performed with R packages “survminer” and “survival” to explore the effect of GBP2 expression on the survival of glioma patients (Liu et al., 2018a). We evaluated overall survival (OS), disease-specific survival (DSS) and progression-free interval (PFI) in glioma patients. Meanwhile, Kaplan-Meier curves were plotted to further speculate the effect of GBP2 expression on the survival of specific variables. To evaluate the predictive power of GBP2 for survival outcomes at different times, we used time-dependent ROC curves. We also plotted the results of multivariate Cox regression analysis by nomogram analysis and visualized the analysis results using R package “rms” and “survival” packages, and performed Calibration analysis at the same time to fit the model established by Cox regression method with the actual situation (Ceccarelli et al., 2016; Liu et al., 2018a).

The CGGA database (http://cgga.org.cn/index.jsp) contains more than 2,000 glioma samples. We obtained three datasets, mRNAse_325 (Bao et al., 2014; Zhao et al., 2017; Zhao et al., 2021), mRNAseq_693 (Zhao et al., 2021; Wang et al., 2015; Liu et al., 2018b), and mRNA_array_301 (Zhao et al., 2017; Fang et al., 2017), from the CGGA database to validate the prognostic value of GBP2 in gliomas. The acquired data were analyzed using R software.

### Gene ontology, kyoto encyclopedia of genes and genomes analysis

Glioma patients were divided into two groups according to the expression of GBP2. Then the two groups were analyzed using the R package “DESeq2” to select differential genes. We used the R package “clusterProfiler” and R package “org.Hs.eg.db” for Gene ontology (GO) and kyoto encyclopedia of genes and genomes (KEGG) analysis of selected differential genes (Yu et al., 2012), and the differential gene screening criteria were |Log2FC| > 1.5, padj < 0.05, resulting in a total of 2,267 differential genes for enrichment analysis.

### Gene set enrichment analysis

The results obtained from the single-gene differential analysis were subjected to Gene set enrichment analysis (GSEA) with the R package “clusterProfiler”. The results were visualized with “ggplot2” (Subramanian et al., 2005).

### Tumor immune infiltration analysis

R package “GSVA” was used to analyze the correlation between GBP2 expression and 24 immune cells. We also analyzed whether the difference between immune cell infiltration and GBP2 expression was statistically significant. We analyzed the relationship between GBP2 expression and immune cell infiltration level with the “estimation” package (Bindea et al., 2013; Hänzelmann et al., 2013). Correlation analysis was performed using Spearman correlation analysis, and *p* < 0.05 was considered statistically significant. Wilcoxon rank sum test was used for differences between normal and tumor groups. Statistical significance: ns, *p* ≥ 0.05; *p < 0.05; **p < 0.01; ***p < 0.001.

### Single-cell analysis

The single-cell data of glioma data (GSE131928) were downloaded from TISCH database (tisch.comp-genomics.org/). The count matrix was processed and visualized using R package “Seurat” with default parameters. Cells were clustered using the first 20 principal components and were annotated according to the information from TISCH databases. Differentially expressed genes were identified by the function “FindMarkers” with min. pct = 0.2. KEGG pathway analysis were conducted by the R package “clusterProfiler.”

### TISIDB: Tumor–immune System Interactions database analysis

The TISIDB database (http://cis.hku.hk/TISIDB/index.php) is a tool of tumor interactions with the immune system that can help predict immunotherapy response (Ru et al., 2019). We analyzed the association between GBP2 and lymphocytes, immunomodulators, immune checkpoints and chemokines. “Rho” values greater than 0.3 and less than -0.3, while satisfying *p* < 0.05 were considered significant.

### Analysis of upstream regulatory mechanism of guanylate binding protein 2

We obtained miRNAs related to GBP2 from three databases: miRTarbasev8.0, Tarbasev8.0 (https://www.networkanalyst.ca/NetworkAnalyst/home.xhtml) (Xia et al., 2013a; Xia et al., 2013b; Zhou et al., 2019) and miRNet 2.0 (https://www.mirnet.ca/miRNet/home.xhtml) (Fan et al., 2016; Chang et al., 2020). LncRNAs related to miRNAs were searched from two databases: miRNet 2.0 and lncbase v3.0 (www.microrna.gr/LncBase) (Karagkouni et al., 2020). The results were presented in Venn diagrams. The GDSC database (https://www.cancerrxgene.org/) uses cell lines to discover potentially sensitive drugs for the treatment of cancer. We used the GDSC database to further analyze GBP2 related valuable clinical therapeutic agents.

### Cell culture of U87 and U251 cells

U87 cells were maintained in Dulbecco’s modified Eagle medium (DMEM) (Gibco), supplied with 10% fetal bovine serum (FBS) (Gibco), 2 mM glutamine and 1% penicillin–1% streptomycin (Gibco). U251 cells were cultured in DMEM (Gibco, CA, United States) medium containing 10% FBS and 1% penicillin–1% streptomycin (Gibco). U87 and U251 cells was purchased from National Infrastructure of Cell line Resource (NICR) (Beijing, China). Cell culture was kept in a 37°C incubator containing 5% CO_2_.

### Guanylate binding protein 2 knockdown

Lipofectamine RNAiMAX (Invitrogen) was used for the transfection of small interfering RNAs (siRNAs) in U87 and U251 cells, following the manufacturer’s protocols. Four hours post-transfection, the culture medium was replaced by fresh medium. After 48 h post-transfection, cells was harvested, and Total RNA was extracted using TRIzol^®^ reagent (Aidlab Biotechnologies Co., Ltd.). The GBP2 knockdown efficiency was detected by RT-qPCR analysis. The sequences of siRNAs against negative control and GBP2 were 5′-UUC​UCC​GAA​CGU​GUC​ACG​U-3′ and 5′-GCC​AGA​ACA​CAC​CCU​AGU​U-3′, respectively.

### miRNAs overexpression

The mimics NC, hsa-miR-26b-5p and hsa-miR-335-5p were obtained from HIPPOBIO (Huzhou, Zhengjiang, China) and were transfected into U87 and U251 cells using Lipofectamine 3000 (Invitrogen), following the manufacturer’s protocols. Four hours post-transfection, the culture medium was replaced by fresh medium. After 48 h post-transfection, cells was harvested, and Total RNA was extracted using TRIzol^®^ reagent (Aidlab Biotechnologies Co., Ltd.). The target gene expression after miRNAs overexpression was detected by RT-qPCR analysis. The sequences of each mimics were listed as follow. Mimics NC sense: 5′-UUU​GUA​CUA​CAC​AAA​AGU​ACU​G-3′; Mimics NC antisense: 5′-CAG​UAC​UUU​UGU​GUA​GUA​CAA​A-3′. hsa-miR-26b-5p mimics sense: 5′-UUC​AAG​UAA​UUC​AGG​AUA​GGU-3′; hsa-miR-26b-5p mimics antisense: 5′-ACC​UAU​CCU​GAA​UUA​CUU​GAA-3′. hsa-miR-335-5p mimics sense: 5′-UAC​AGU​ACU​GUG​AUA​ACU​GAA-3′; hsa-miR-335-5p mimics antisense: 5′-UUC​AAG​UAA​UUC​AGG​AUA​GGU-3′.

### Quantitative real-time PCR

Total RNA extraction, reverse transcription and real-time PCR (RT-qPCR) experiments were conducted according the protocols as described previously (Ren et al., 2022). The following primers were used: GBP2 forward, 5′-GAT​TGG​CCC​GCT​CCT​AAG​AA-3′ and reverse, 5′-TTG​ACG​TAG​GTC​AGC​ACC​AG-3’; HLA-DRA forward, 5′-ATA​CTC​CGA​TCA​CCA​ATG​TAC​CT-3′ and reverse, 5′-GAC​TGT​CTC​TGA​CAC​TCC​TGT-3’; B2M forward, 5′-GAG​GCT​ATC​CAG​CGT​ACT​CCA-3′ and reverse, 5′-CGG​CAG​GCA​TAC​TCA​TCT​TTT-3′; HLA-DPB1 forward, 5′-ATT​CTG​CCC​GGA​GTA​AGA​CAT-3′ and reverse, 5′-TCG​TTG​AAC​TTT​CTT​GCT​CCT​C-3′; HLA-A forward, 5′-AAA​AGG​AGG​GAG​TTA​CAC​TCA​GG-3′ and reverse, 5′-GCT​GTG​AGG​GAC​ACA​TCA​GAG-3′; HLA-DMA forward, 5′-ACA​AAG​AGT​TCT​GCG​AGT​GGA-3′ and reverse, 5′-ACT​TCA​GCG​ATA​GGA​AAC​CCT​C-3′; HLA-B forward, 5′-CAG​TTC​GTG​AGG​TTC​GAC​AG-3′ and reverse, 5′-CAG​CCG​TAC​ATG​CTC​TGG​A-3′; CD74 forward, 5′-CCG​GCT​GGA​CAA​ACT​GAC​A-3′ and reverse, 5′-GGT​GCA​TCA​CAT​GGT​CCT​CTG-3′; HLA-E forward, 5′-TTC​CGA​GTG​AAT​CTG​CGG​AC-3′ and reverse, 5′- GTC​GTA​GGC​GAA​CTG​TTC​ATA​C-3′; HLA-DRB5 forward, 5′-CGG​GGT​TGG​TGA​GAG​CTT​C-3′ and reverse, 5′-AAC​CAC​CTG​ACT​TCA​ATG​CTG-3′; HLA-DRB1 forward, 5′-CGG​GGT​TGG​TGA​GAG​CTT​C-3′ and reverse, 5′-AAC​CAC​CTG​ACT​TCA​ATG​CTG-3′; HLA-DPA1 forward, 5′-CAA​GGC​GGA​CCA​TGT​GTC​AA-3′ and reverse, 5′-GTG​GTT​GGA​ACG​CTG​GAT​CA-3′; CCL2 forward, 5′-CAG​CCA​GAT​GCA​ATC​AAT​GCC-3′ and reverse, 5′-TGG​AAT​CCT​GAA​CCC​ACT​TCT-3′; CCL5 forward, 5′- CCA​GCA​GTC​GTC​TTT​GTC​AC-3′ and reverse, 5′- CTC​TGG​GTT​GGC​ACA​CAC​TT-3′; CXCL10 forward, 5′- GTG​GCA​TTC​AAG​GAG​TAC​CTC-3′ and reverse, 5′- TGA​TGG​CCT​TCG​ATT​CTG​GAT​T-3′; β-actin forward, 5′-CAT​GTA​CGT​TGC​TAT​CCA​GGC-3′ and reverse, 5′-CTC​CTT​AAT​GTC​ACG​CAC​GAT-3′. 2^−ΔΔ^CT was used to represent the expression.

### Statistical analysis

Statistical analysis was performed using R version. The Wilcoxon rank sum test was used for statistics of differences in gene expression in pan-cancer. The Wilcoxon rank sum test was used for comparisons between two groups and the Kruskal-Wallis test for multiple group comparisons. The survival package was used for KM, univariate, and multivariate Cox regression analyses to calculate the risk ratio, *p* value, and risk confidence interval. The *p* value of KM survival curves was calculated by the log-rank test. GSVA package was used for immune correlation analysis and Spearman correlation analysis was used to determine whether there was a correlation between the two groups. *p* < 0.05 was considered as statistically significant.

For qPCR analysis, data were presented as mean ± standard deviation, and analyzed by GraphPad Prism 8.0 software. Student’s *t*-test was applied to compare the difference between two groups. One-way ANOVA followed by Tukey’s post hoc test was applied to compare the difference among more than two groups. *p* < 0.05 was considered as statistically significant.

## Results

### Guanylate binding protein 2 showed higher expression in glioma

**FIGURE 1:**
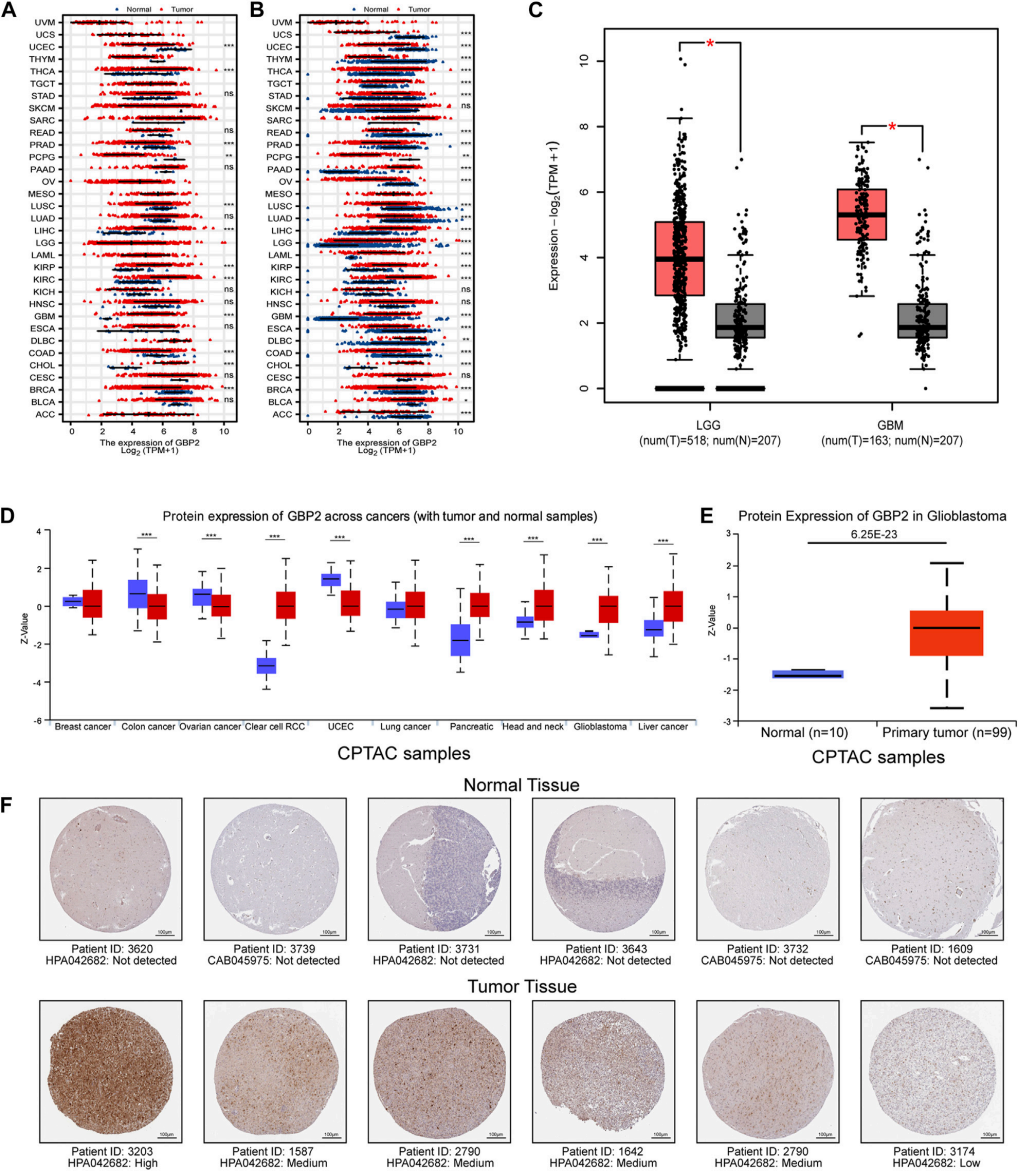
GBP2 was highly expressed at different levels in glioma. **(A)** GBP2 expression in GTEx normal tissue and TCGA tumor tissue at RNA level. **(B)** GBP2 expression between TCGA tumor tissue and TCGA adjacent normal tissue at RNA level. Ns, *p* ≥ 0.05; *p < 0.05; **p < 0.01; ***p < 0.001. **(C)** The RNA levels of GBP2 between tumors and normal samples in LGG and GBM samples (GEPIA database). The red asterisk means the *p*-value is less than 0.01. **(D)** The protein level of GBP2 between tumors and normal tissue across cancer types in Ualcan database. **(E)** The protein level of GBP2 between glioma and normal samples in Ualcan database. **(F)** Immunohistochemistry was used to validate the high protein levels of GBP2 in glioma tissues (HPA database). *p < 0.05.

We analyzed the change of GBP2 among 33 cancer types by comparing tumor tissue with normal tissue and adjacent normal tissue (Figures 1A,B). GBP2 has consistently higher expression in four tumors, including GBM, CHOL, KIRC and KIRP, while has consistently lower expression in four tumors, including BRCA, COAD, LUSC and UCEC. In particular, GBP2 showed the highest fold change in gliomas among other cancer types (Figure 1C).

We further analyzed GBP2 at the protein level in UALCAN database. GBP2 is highly expressed in GBM, KIRC, HNSC, PAAD, LIHC and lowly expressed in COAD, OV, UCEC (Figures 1D,E). In addition, GBP2 showed higher expressions in tumor tissue from glioma patients than in normal brain tissue in the Human Protein Atlas database (Figure 1F). These consistent results suggest that glioblastoma has higher GBP2 protein levels than normal tissue at both RNA and protein level. Therefore, we further investigated the role of GBP2 in gliomas.

### Poor clinicopathological features are associated with guanylate binding protein 2 expression in glioma

**FIGURE 2:**
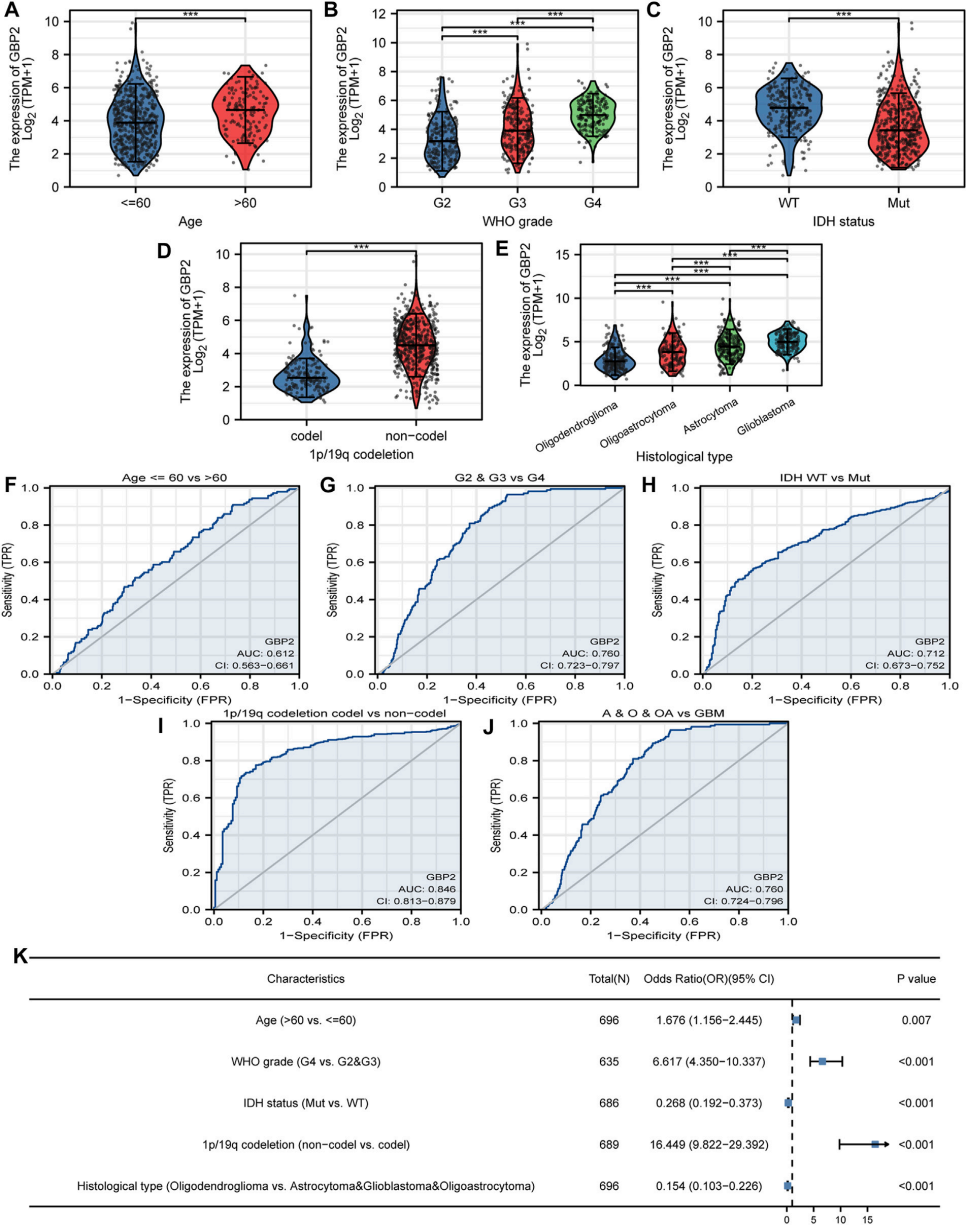
GBP2-related clinicopathological features in glioma. GBP2 expression in glioma clinically relevant features including age **(A)**, WHO grade **(B)**, IDH status **(C)**, 1p/19q cedeletion **(D)**, histological type **(E)**. ROC curves show the diagnostic efficacy of GBP2 expression for age **(F)**, WHO grade **(G)**, IDH status **(H)**, 1p/19q cedeletion **(I)**, histological type **(J)**. **(K)** Logistic regression analysis of clinicopathological parameters and GBP2 expression. ***p < 0.001.

We divided glioma patients into two groups according to GBP2 expression, to analyze the relationship between clinicopathological features and GBP2 expression. The result showed that GBP2 expression was associated with age, WHO grade, IDH status, 1p/19q co-deletion, and histological type (Figures 2A–E). Also, GBP2 was able to significantly distinguish these features using receiver operating characteristic curve (ROC) (Figures 2F–J). Furthermore, logistic regression analysis demonstrated that GBP2 expression was associated with clinicopathological features (Figure 2K). GBP2 expression showed relatively higher odds ratios with 1p/19q co-deletion, and higher WHO grade. These malignant characteristics in patients with higher GBP2 expression suggested the significant role of GBP2 in gliomas.

### Prognosis analysis of guanylate binding protein 2 in glioma

**FIGURE 3:**
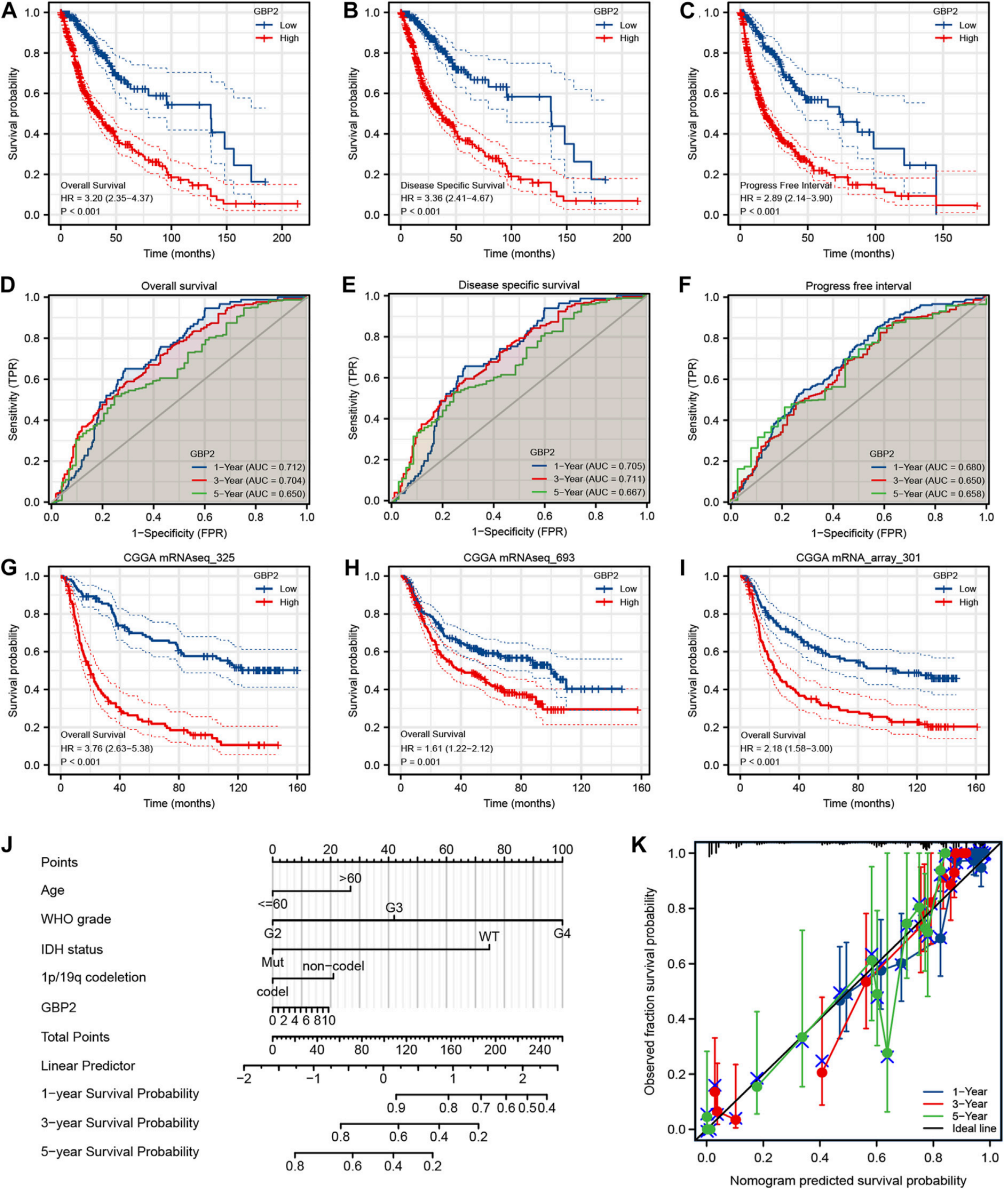
Analysis of the prognostic value of GBP2 using multiple databases. Kaplan-Meier survival analysis for OS **(A)**, DSS **(B)** and PFI **(C)** in glioma patients from the TCGA database. Time-dependent ROC curves of GBP2 expression for OS **(D)**, DSS **(E)** and PFI **(F)**. Blue, 1-year; red, 3-year; green, 5-year. **(G–I)** Kaplan-Meier survival analysis for OS using CGGA datasets. **(J)** Nomogram model for glioma patients. **(K)** Calibration curves of nomogram.

Kaplan-Meier analysis indicated that high expression of GBP2 was significantly associated with worse overall survival (OS), disease specific survival (DSS) and progress-free interval (PFI) in glioma patients (Figures 3A–C). Further analysis of glioma subgroup revealed that high GBP2 expression was significantly associated with high-grade gliomas patients (Supplementary Figures S1A–C). The reliability of GBP2 in predicting the viability of glioma patients was determined by time-dependent ROC curves (Figures 3D–F). These results indicated that GBP2 had potential in monitoring survival. The glioma datasets from the CGGA database also verified that high GBP2 expression was associated with poor prognosis in glioma patients (Figures 3G–I).

In addition, we constructed a prognostic nomogram based on GBP2 expression and clinicopathological factors, in order to predict the survival risk of individual patients (Figure 3J). Consistently, the calibration curve of the prognostic nomogram showed a high concordance between prognosis and actual survival (Figure 3K).

Multivariate Cox regression analysis of OS and PFI showed that WHO grade, and IDH status were independent prognostic factors (Supplementary Figures S1D,F). In addition, multivariate Cox regression of DSS showed that GBP2 expression, IDH status and WHO grade were independent prognostic indicators (Supplementary Figure S1E). Altogether, these data suggested that GBP2 was significantly correlated with worse prognosis in glioma.

### Immune-related characteristics of guanylate binding protein 2 in glioma

**FIGURE 4:**
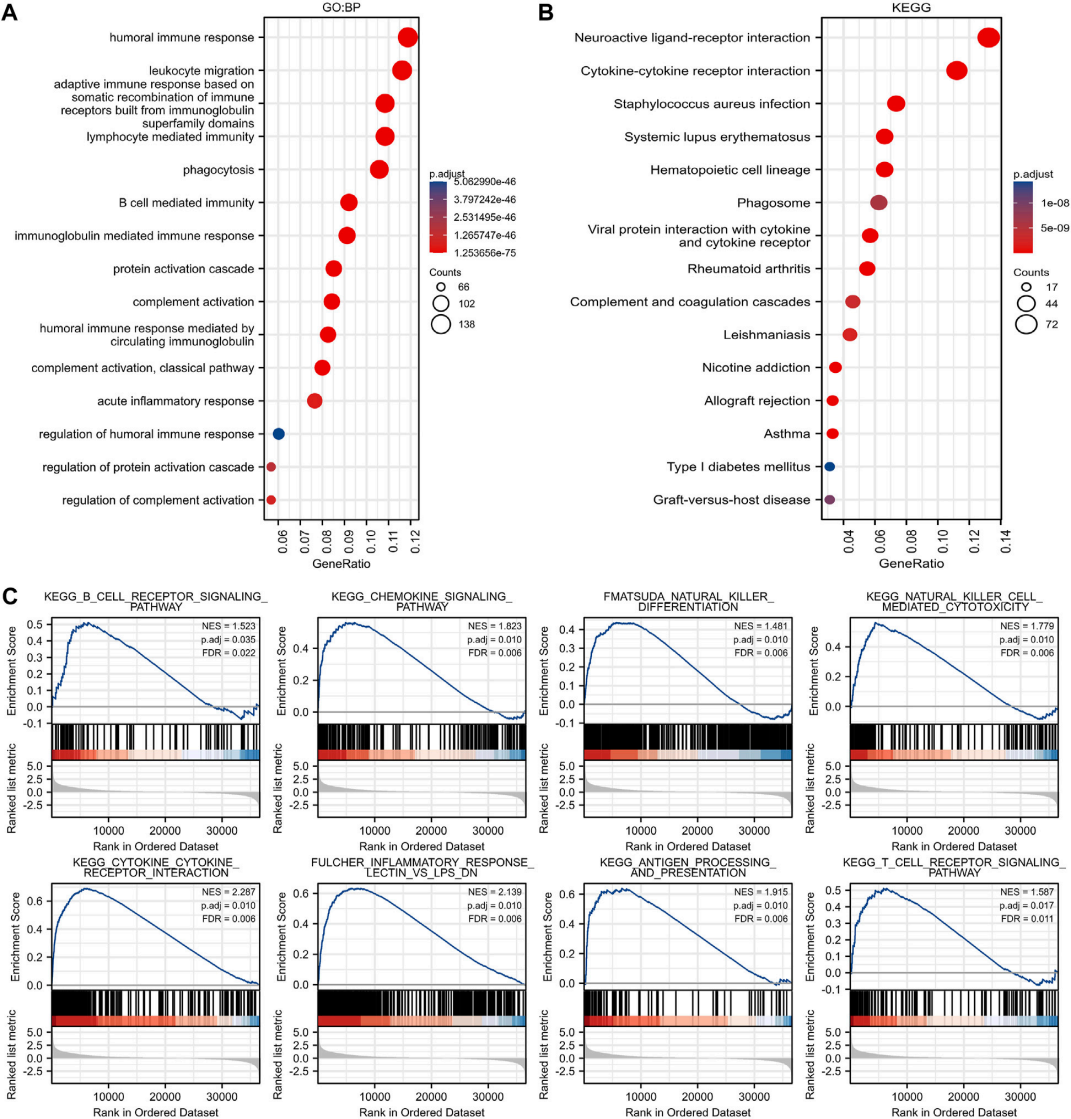
Immune-related pathways were enriched in GBP2-related genes. **(A)** The biological processes of GBP2-associated genes in the GO database are mainly associated with the human immune response. **(B)** The pathway enrichment of GBP2-associated genes in the KEGG database is mainly associated with immune processes such as neuroactive ligand-receptor interaction, cytokine-cytokine receptor interaction. **(C)** GSEA analysis of GBP2-associated genes revealed differential genes mainly associated with immune cell receptor signaling pathway, chemokine signaling pathway and other immune-related pathways.

GBP2-related functional enrichment analysis was performed in Gene Ontology (GO), Kyoto Encyclopedia of Genes and Genomes (KEGG) database. The GBP2 expression pattern was mainly associated with human immune response, leukocyte migration and adaptive immune response in GO biological process, was linked with external side of plasma membrane, immunoglobulin complex and synaptic membrane in GO cellular component, and was related with antigen binding, receptor ligand activity and passive transmembrane transporter activity in GO molecular function (Figure 4A, Supplementary Figures S2A,B). The KEGG analysis results included “Neuroactive ligand-receptor interaction”, “Cytokine-cytokine receptor interaction”, “*Staphylococcus aureus* infection”, “phagosome”, “Antigen processing and presentation” (Figure 4B). Gene Set Enrichment Analysis (GSEA) analysis also revealed immune related signaling pathways (Figure 4C, Supplementary Figures S2C,D). Altogether, these results indicate that GBP2 expression is likely to affect immune-related pathways in patients with glioma.

### Guanylate binding protein 2 expressions were correlated to immune infiltration in glioma

**FIGURE 5:**
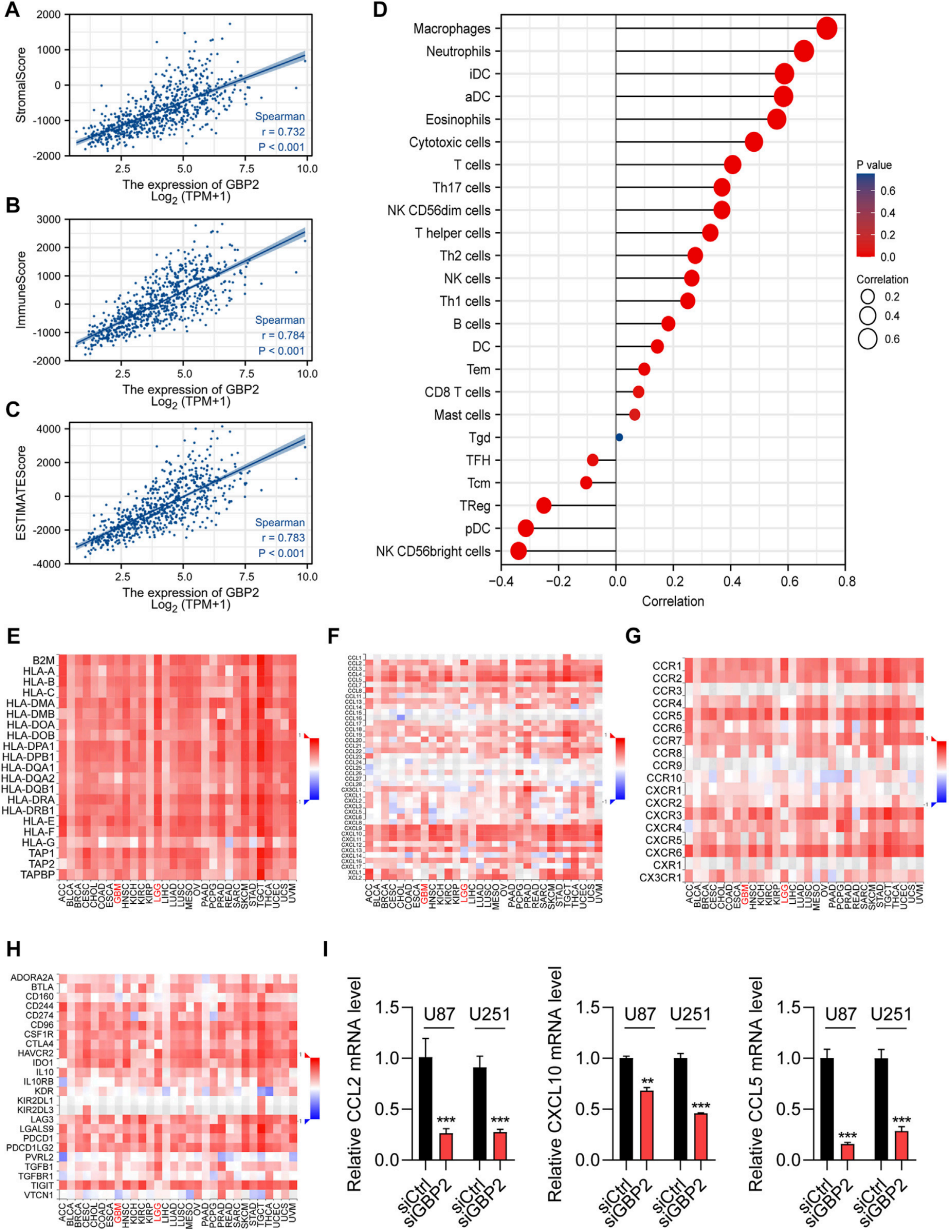
GBP2 showed significant correlations with immune infiltration in the glioma microenvironment. **(A–C)** The Spearman correlations of GBP2 expression with stromal score **(A)**, immune score **(B)**, ESTIMAT score**(C)**. **(D)** The lollipop graph shows immune cell associations with GBP2 expression. **(E–G)** The associations of GBP2 expression with antigen presentations genes **(E)**, chemokines **(F)** chemokine receptors **(G)**, and immune checkpoints **(H)**. **(I)** qRT-PCR analysis of chemokines genes in U87 and U251 cells transfected with siCtrl and siGBP2. The expression of indicated genes was adjusted to the expression of β-actin. **p < 0.01. ***p < 0.001.

Immune score, stromal score and ESTIMATE score were found to be positively correlated with GBP2 expression, and immune scores showed the highest coefficients (Figures 5A–C).

We then explored the correlation of GBP2 expression with immune cells in gliomas. GBP2 was associated with 23 of 24 immune cells. Positive correlations were showed in myeloid cells, including macrophages, neutrophils, iDCs, aDCs and Eosinophils. Negative correlations were showed in Treg, Plasmacytoid DC and NK CD56^bright^ cells (Figure 5D). Consistently, 20 immune cells were significant correlated with GBP2 expression using enrichment score (Supplementary Figure S3A). The GBP2 was positively correlated with macrophages, neutrophils, iDCs, aDCs, and Eosinophils (Supplementary Figure S3B), while the infiltration of NK CD56^bright^ cells decreased with increasing GBP2 expression (Supplementary Figure S3B).

We next explore the associations between GBP2 expression and key immunomodulators. We found the significant correlations of GBP2 expression with MHCs (HLA-E, TAPBP and HLA-DMB), chemokines and cytokine receptors (CCL2, CCL5, CXCL10, CCR1 and CCR5), and immune checkpoints (CD274, PDCD1, PDCD1LG2 and HAVCR2) (Figures 5E–H, Supplementary Figures S4A–D). Chemokines play an influential regulatory role in T cell infiltration. Moreover, we validated the significant correlations between GBP2 expression and chemokines in U87 and U251 cells. qRT-PCR results showed that GBP2 knockdown suppressed the expression of CCL2, CCL5 and CXCL10 (Figure 5I). In brief, these data suggested that GBP2 played an important role in glioma immune microenvironment, and may be involved in immune regulation.

### Malignant cells with high guanylate binding protein 2 expressions showed higher antigen presentation in glioma patients at single-cell levels

**FIGURE 6:**
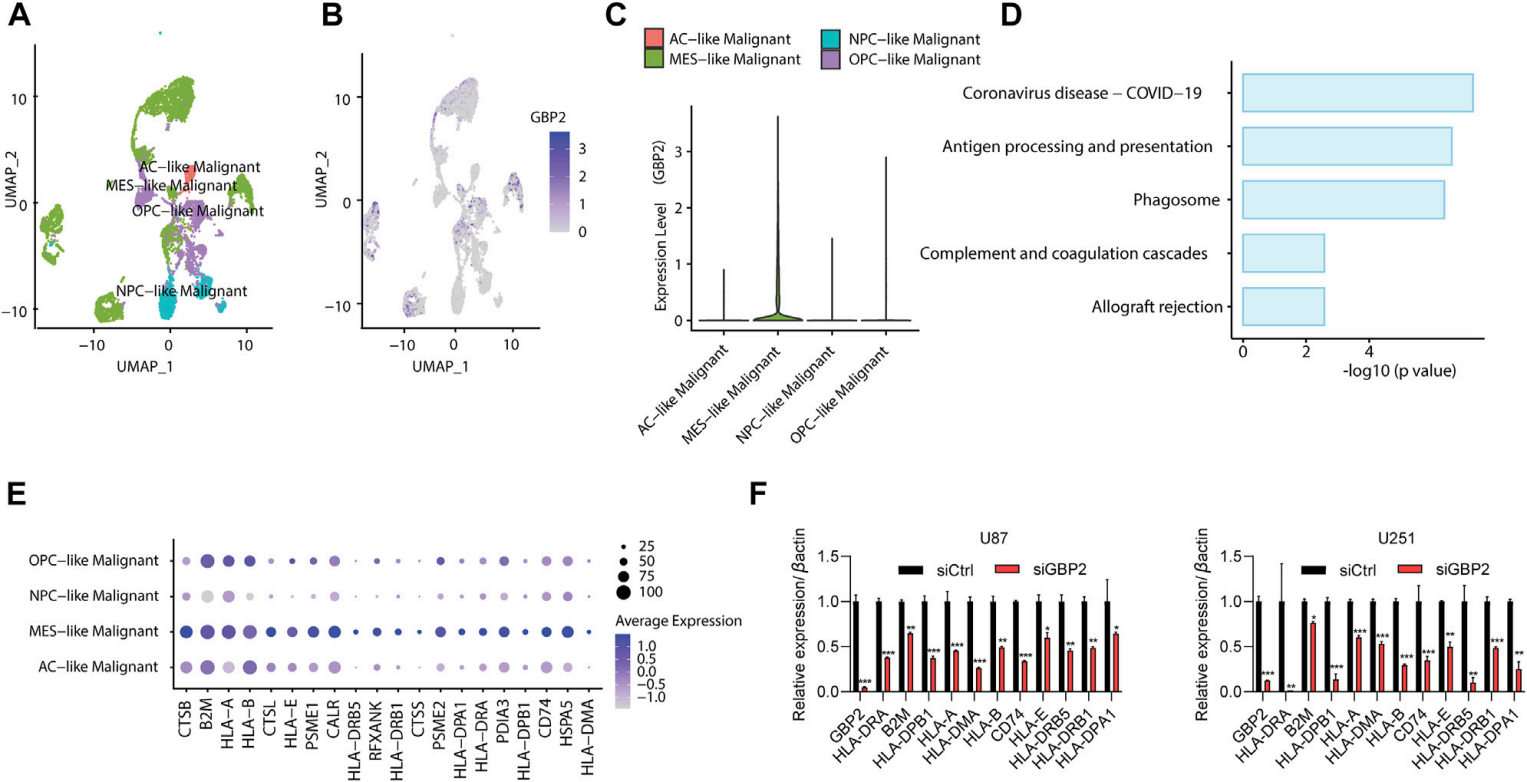
GBP2 expression in glioma microenvironment. **(A)** UMAP plot showing the subtype of malignant cells in glioma patients (GSE131928). **(B,C)** UMAP plot **(B)** and violin plot **(C)** showing the GBP2 expression among glioma subtypes. **(D)** KEGG pathway enrichment of highly expressed genes in GBP2-high (MES-like) malignant cells. **(E)** Dot plot showing antigen presentation genes across four subtype of glioma cells. **(F)** qRT-PCR analysis of indicated genes in U87 and U251 cells transfected with siCtrl and siGBP2. The expression of indicated genes was adjusted to the expression of β-actin. *p < 0.05. **p < 0.01. ***p < 0.001.

To further explore the effect of GBP2 expression on tumor immune microenvironment, we examined the malignant cells in glioma patients (GSE131928). By comparing GBP2 expression among different glioma subtypes, we found GBP2 has the highest expression in MES-like cancer cells (Figures 6A–C).

Next, we identified the differentially expressed genes between GBP2-high malignant cells (MES-like cancer cells) and other cells. The KEGG pathway analysis revealed that GBP-high cells were enriched in immune-related pathways, such as antigen processing and presentation, complement and coagulation cascade, and coronavirus disease-COIV-19 (Figure 6D). In particular, MES-like cancer cells showed higher expression of antigen presentation genes (Figure 6E). This is consistent with the positive correlations in TISIDB database (Figure 5E).

We further validated the significant correlations between GBP2 expression and antigen presentation genes in U87 and U251 cells. qRT-PCR results showed that GBP2 knockdown suppressed the expression of HLA-DRA, B2M, HLA-DPB1, HLA-A, HLA-DMA, HLA-B, CD74, HLA-E, HLA-DRB5, HLA-DRB1 and HLA-DPA1 (Figure 6F).

### Guanylate binding protein 2 were regulated by non-coding RNAs in glioma

**FIGURE 7:**
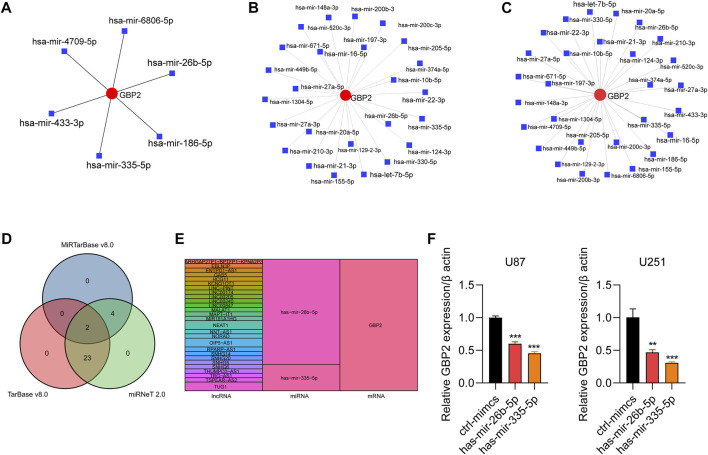
The role of noncoding RNAs in regulating GBP2. **(A–C)** GBP2-related miRNAs in miRTarbase database **(A)**, Tarbase database **(B)** and miRNet database **(C)**. **(D)** Venn diagram showing overlapping miRNAs that are predicted to target GBP2 in three databases, including miRTarBase, TarBase, miRNet. **(E)** Sankey plot showing regulatory network among lncRNAs, miRNAs, and mRNAs. The colored bands visualize the relationship between regulators and their predicted targets. **(F)** qRT-PCR analysis of GBP2 in U87 and U251 cells transfected with mimics NC, mimics miR-26b-5p and mimics miR-335-5p. The expression of GBP2 was adjusted to the expression of β-actin. **p < 0.01, ***p < 0.001.

We further analyzed miRNAs, lncRNAs related to GBP2. We screened 6, 25 and 29 miRNAs related to GBP2 from three databases: miRTarbasev8.0, Tarbasev8.0 and miRNet 2.0, respectively (Figures 7A–C). Two miRNAs hsa-mir-26b-5p and hsa-mir-335-5p were selected by intersecting miRNAs using Venn diagram (Figure 7D). In addition, we obtained lncRNAs related to the two selected miRNAs in two databases (miRNet 2.0 and lncbase v3.0) (Supplementary Figures S5A–D). The GBP2-related ceRNA regulatory network revealed the potential regulatory mechanism of non-coding RNA for GBP2 expression in glioma (Figure 7E).

To further validate the regulatory function of non-coding RNA in GBP2 expression, we overexpressed miR-26b-5p and miR-335-5p in U87 and U251cells and subjected the cells to qRT-PCR analysis of GBP2. The results showed that both miR-26b-5p and miR-335-5p overexpression downregulated the mRNA expression of GBP2 in U87 and U251 cells (Figure 7F).

## Discussion

Gliomas have a poor prognosis and are prone to recurrence because it is difficult to completely identify tumor cells by surgery and drug treatment (Galstyan et al., 2019; Zhang et al., 2020). Immunotherapy offers new solutions for glioma treatment. GBP2 promotes glioblastoma invasiveness through the Stat3-mediated immune pathway (Yu et al., 2020). We therefore investigated the tole of GBP2 in tumor immune microenvironment. GBP2 was found to be highly expressed in several tumors including gliomas (glioblastoma multiforme and low-grade gliomas). GBP2 was further validated at the protein level using the HPA database and Ualcan database. The results demonstrated that GBP2 expression was elevated in glioma tissues.

We investigated the clinical significance of GBP2 in glioma. GBP2 was correlated with age, WHO grade, IDH status, 1p/19q codeletion, histological type, OS, DSS and PFI, but not gender. Logistic regression also illustrated an association between GBP2 and glioma clinical characteristics. Further ROC curves were performed for GBP2 to analyze its diagnostic efficacy for clinical features. The results showed that GBP2 had good diagnostic ability in glioma, WHO grade (G2 vs. G4, G3 vs. G4), 1p/19q codeletion. IDH status, and histological type (oligodendroglioma vs. astrocytoma, oligodendroglioma vs. glioblastoma, and oligodendroglioma vs. glioblastoma). The analysis demonstrated that the expression of GBP2 has an important impact on clinicopathological features in glioma.

We further examined the prognostic significance of GBP2 for glioma. In KM analysis of WHO grades, we found that high GBP2 expression in WHO grades (G3 and G4) predicted worse survival, which indicated high expression of GBP2 also predicts poor survival outcomes in high-grade gliomas. Time-dependent ROC curves also demonstrated that GBP2 has predictive power in glioma prognosis. In multivariate Cox analysis of DSS, high GBP2 expression was an independent prognostic factor for glioma survival. Prognostic nomograms and Calibration curves indicate that the Cox regression analysis model has significant accuracy.

To analyze the function of GBP2 and the possible biological pathways involved, we performed functional enrichment analysis of GBP2-related genes. GO analysis and GSEA analysis revealed that GBP2 was related to immunity. KEGG analysis also included “Antigen processing and presentation,” “Neuroactive ligand-receptor interaction,” “Cytokine-cytokine receptor interaction” and “*Staphylococcus aureus* infection.” It suggested that GBP2 may be linked with the immune response process. Multiple pathways showed that GBP2 was associated with tumor immunity.

We next explored the role of GBP2 in tumor immunity of glioma. GBP2 was positively correlated with macrophages, neutrophils, iDCs, aDCs, and eosinophils. These results were also confirmed by immune cell enrichment analysis and ESTIMATE analysis. We also utilized the TISIDB database for immunoassays, the result indicated that GBP2 was positively correlated with macrophage, iDCs. GBP2 was positively correlated with antigen presentation molecules (HLA_E, HLA_DBM), immune checkpoints (CD274, HAVCR2), cytokines (CCL2, CXCL10) and cytokine receptors (CCR1, CCR5). Single-cell analysis revealed that GBP2 were highly expressed in MES-like glioma subtypes, which showed the high antigen presentation capabilities. These integrated analyses suggest that GBP2 glioma is involved in immune regulation and might be a valuable immunotherapeutic target.

Further analysis of noncoding regulators revealed GBP2-involved ceRNAs network. The hsa-mir-26b-5p and hsa-mir-335-5p were found to regulate GBP2 expression, and were also related to several lncRNAs. Therefore, GBP2-involved non-coding network provide insights into the noncoding-mediated mechanisms in glioma prognosis and therapy.

## Data Availability

The original contributions presented in the study are included in the article/Supplementary Material, further inquiries can be directed to the corresponding author.
