# Supplementary material for: GBP2 as a potential prognostic predictor with immune-related characteristics in glioma
Source: Front Genet. 2022 Sep 16;13:956632. doi: 10.3389/fgene.2022.956632 (PMC9523311; doi:10.3389/fgene.2022.956632)
Supplement: Supplementary file 1 [file DataSheet1.docx]

**Figure S1**


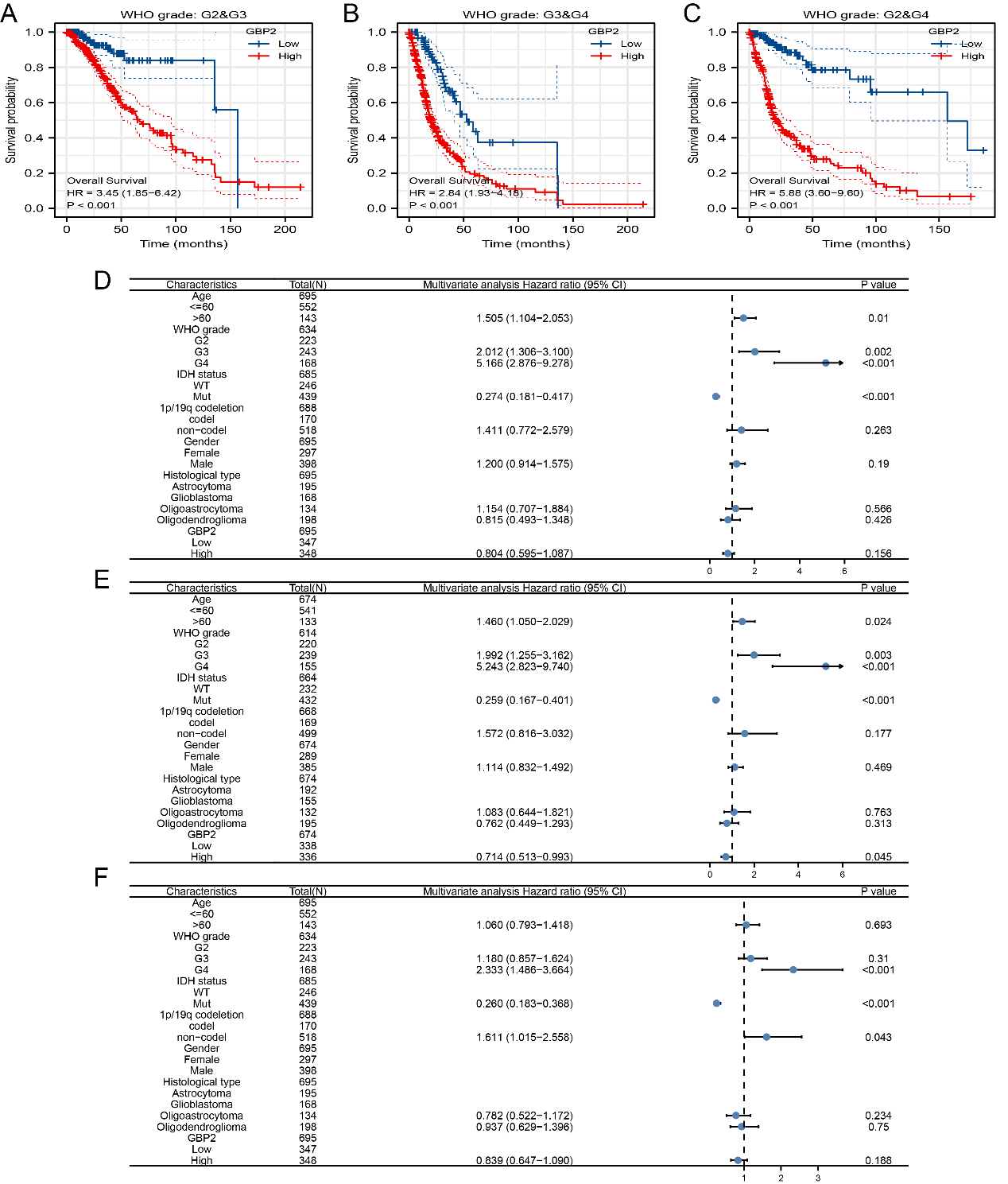


**Figure S1. Identification of GBP2-related prognostic features. (A-C)** Kaplan-Meier survival analysis of high-grade gliomas for OS **(A),** DSS **(B)** and PFI **(C)**. **(D-F)** Multivariate Cox regression analysis of OS **(D),** DSS **(E)** and PFI **(F)** with GBP2 expression. P<0.05 was considered statistically significant.

**Figure S2**

**Figure S2. Pathway Enrichment of GBP2-related genes. (A)** Cellular component enrichment of GBP2-related genes in GO database. **(B)** Molecular function enrichment of GBP2-related genes in GO database. **(C-D)** Immune-related pathways were enriched in GBP2-related genes by GSEA analysis.

**Figure S3**


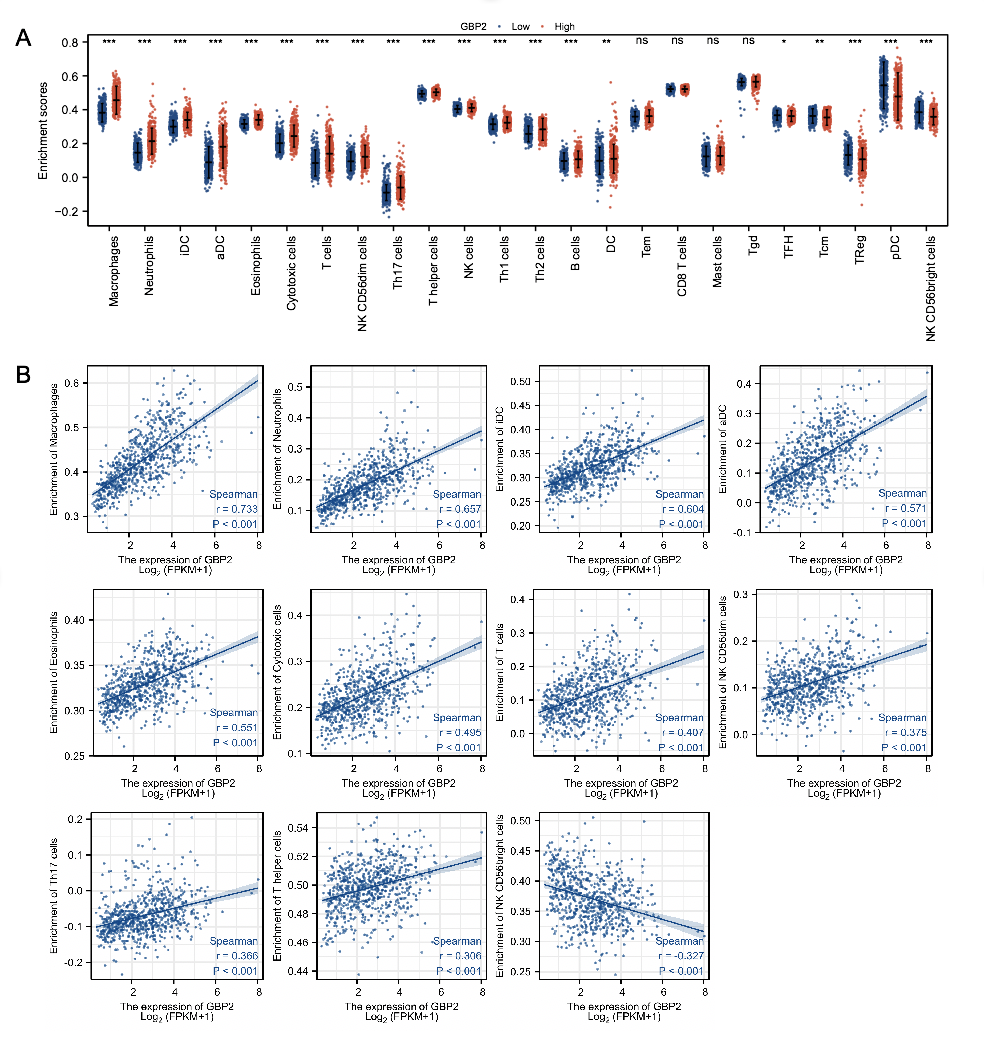


**Figure S3.** **(A)** Box plot illustrating high and low GBP2 and immune cell infiltration levels. ns, p≥0.05; *, p< 0.05; **, p<0.01; ***, p<0.001. **(B)** The correlation (|r|>0.3) between infiltration levels of immune cell and GBP2 expression in glioma.

**Figure S4**

**
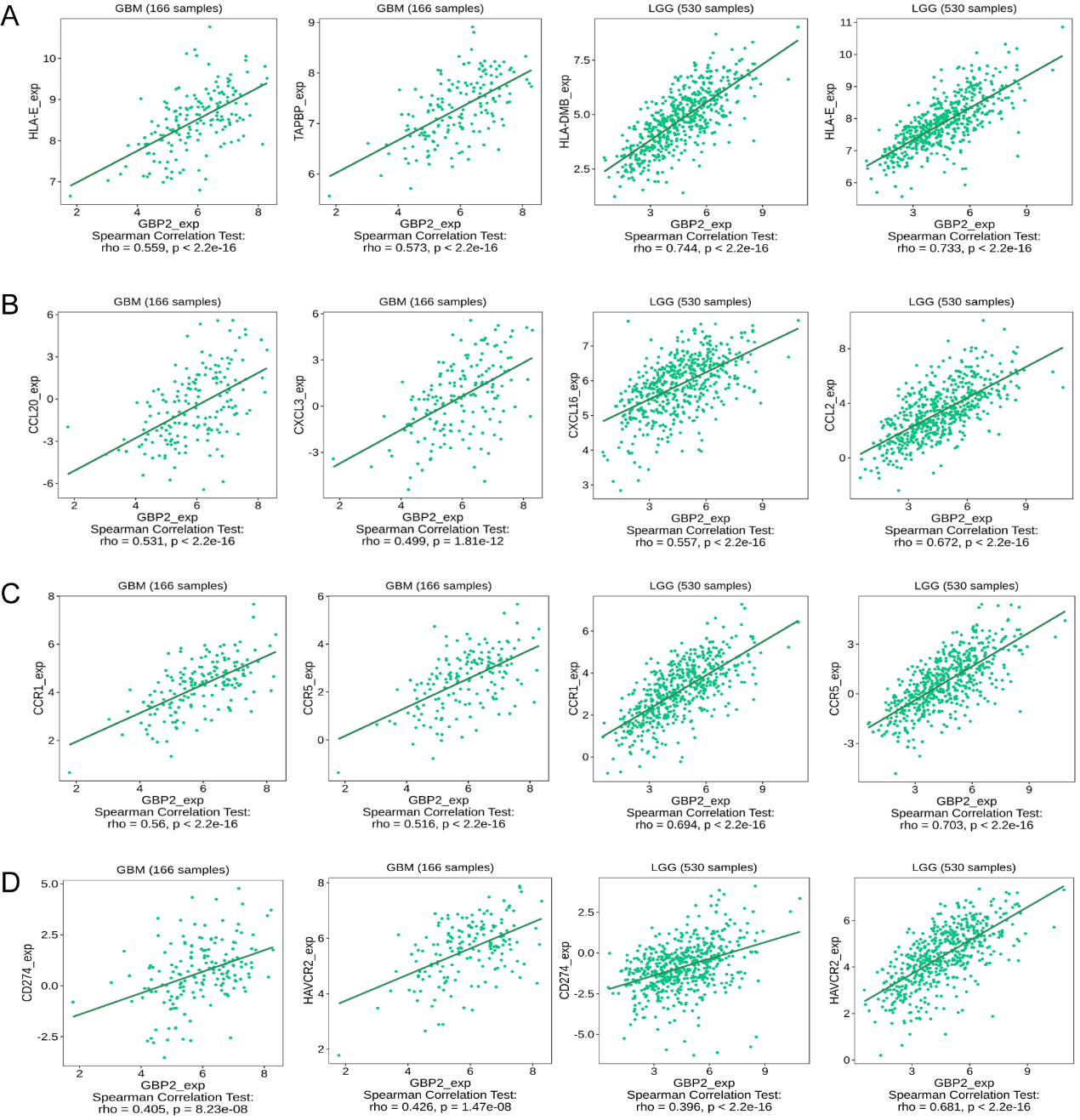
**

**Figure S4. Identification of GBP2-related immune features in the TISIDB database.** **(A-C)** Correlations between GBP2 expression and MHC molecules **(A),** chemokines **(B)** chemokine receptors **(C)** and immune checkpoints **(D)** in gliomas.

**Figure S5**

**Figure S5. Identification of GBP2-related lncRNAs. (A)** has-mir-335-5p related lncRNAs in miRNet 2.0 and lncbase v3.0 database. **(B)** has-mir-26b-5p related lncRNAs in miRNet 2.0 and lncbase v3.0 database. **(C)** Venn diagram shows intersection of has-mir-335-5p related lncRNAs in two lncRNA databases. **(D)** Venn diagram shows intersection of has-mir-26b-5p related lncRNAs in two lncRNA databases.
